# Prediction of Melting Points of Chemicals with a Data Augmentation-Based Neural Network Approach

**DOI:** 10.1021/acsomega.5c00205

**Published:** 2025-06-03

**Authors:** Lea E. Austermeier, Karsten Voigt, Alexander Böhme, Nadin Ulrich

**Affiliations:** † Department of Exposure Science, 28342 Helmholtz Centre for Environmental Research—UFZ, Permoserstrasse 15, Leipzig D-04318, Germany; ‡ PAULY, Theresienstrasse 50, Leipzig D-04129, Germany

## Abstract

The melting point (MP) of a chemical is an important physicochemical property that characterizes the transition from a solid to a liquid state. The MP is a key parameter in molecular design and relevant in many fields such as drug design and environmental science. Therefore, an accurate prediction of the MP is of huge interest. Here, we develop two graph convolutional neural network (GNN) models for the prediction of the MP: one where we do not apply a data augmentation strategy and one where we apply a data augmentation strategy. The models were developed on a data set containing 28,645 chemicals, where we removed duplicates and data points labeled as faulty. Then we split the data set into training, validation, and test sets. The model was trained on this initial data set and on a higher curated data set. Based on the data augmentation, we could enlarge the number of neurons in each of the two hidden layers in the GNN and reinforce the representation of large and complex molecules. We compared the influence of the curation step and the data augmentation and found that the curation step had no significant influence on the model performance, while the model could be improved by the application of data augmentation. With a consensus model, we achieved an rmse of 35.4 °C.

## Graphical abstract

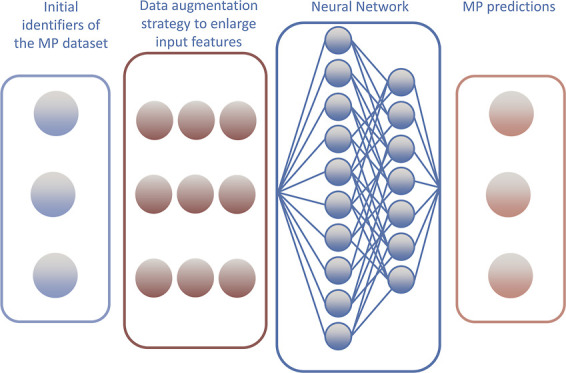

## 1 Introduction

The melting point (MP) is an important physicochemical property that describes the temperature at which a compound changes from the solid state to liquid state. It depends on the enthalpy, which is the energy due to intermolecular attractions and the formation of a crystal lattice, and on the entropy, which is the energy gained by the increased disorder.^1^ In experimental practice, the MP is often used to determine the purity of compounds.^2^

There are different methods for determining the experimental MP, such as differential scanning calorimetry (DSC) and the capillary tube method. With the DSC method, thermal transitions can be determined by gradually heating a sample and measuring the energy needed to maintain the temperature compared to a reference or measuring the temperature difference between the reference and sample.^3^ For the capillary tube method, the sample is introduced into a capillary, and the temperature is gradually increased until the sample is visibly melted completely.^4^ This method is typically used to determine the MPs of crystalline organic compounds or fats.^5^ Even though pure compounds theoretically have one definite MP, the experimental MP is not distinct due to different restrictions. Typical restrictions are impurities of the compound and measuring errors since the experiments are often conducted only once. Furthermore, the compounds can decompose and form liquid decomposition products.^6^ However, it is not always known if a molecule decomposes during melting.^7^

The experimental determination of MPs is costly and time-intensive. However, the knowledge about the MPs of chemicals is of great interest in different fields, such as molecular design and risk assessment. It needs to be provided in REACH dossiers, e.g., as it indicates the physical state of the chemical and allows us to assess potential exposure of humans. Further, the environmental behavior is impacted by the physical state as well. Therefore, an accurate prediction of the MPs could provide a remedy.^8,9^

The determination of the MP from the chemical structure is very complex. While the boiling point depends on only the pairwise interactions of the molecules, the MP is further dependent on interactions in the crystal lattice. Even for the same molecule, the MP can differ in crystalline or amorphic state.^10^ The incorporation of a molecule into the crystal lattice is influenced by molecular properties, such as chirality, eccentricity, symmetry, and flexibility. Generally, it can be stated that with an increasing molar mass, the MP increases.

Furthermore, symmetric molecules show higher MPs than asymmetric molecules, since symmetric molecules form crystalline structures more easily. Additionally, planar molecules show a higher MP because they can be packed more efficiently in the lattice than molecules with a high eccentricity. For chiral molecules, the MP of racemic mixtures can differ from the MP of the respective enantiomers due to different packing efficiency. The flexibility of a molecule is dependent on the bonds. Single bonds are more flexible, and the molecule can align to reach the maximum intermolecular dispersion. Furthermore, free rotation can increase the MP by achieving the optimal conformation for packing. Since the modeling of the crystal structure is still very challenging, there are several approaches to predict the MP based on experimental MP data.^10^ When developing such models based on experimental data, it must be considered that the model can only perform as well as the accuracy of the experimental MPs.^7^ Various quantitative structure–property relationships are around to predict MPs, some are based on group contribution methods or topological descriptors, e.g.,^11^ others are based on molecular simulations,^12^ thermodynamic calculations,^6^ artificial neural networks,^13^ and k-nearest neighbor algorithms.^2^ Nigsch et al. created a model based on the k-nearest neighbor approach. They used a data set with 4,119 molecules and the Bergström data set containing of 277 druglike chemicals.^2^ With a combination of 2D and 3D descriptors an rmse of 46.3 °C was obtained on the test set when splitting the Bergström data set in training and test set.^2^ Karthikeyan et al. developed an artificial neural network approach based on 4,173 compounds using 2D descriptors, which they chose by principal component analysis.^13^ With their approach they reached an rmse of 41.4 °C on the external Bergström data set. Carrera et al. tested generical molecular maps of atom-level properties combined with a random forest algorithm for the MP prediction.^14^ The model was developed on 2,600 data points obtained from the highly curated Bradley data set. With this approach, an rmse of 44.0 °C was achieved. Chen et al. tested a deep learning approach based on knowledge-infused molecular graphs on a data set for melt-castable energetic chemicals, including 29,713 compounds with melting points between 343 and 393 K in the model development.^15^ First, the model processes compounds as molecular graphs in a message-passing neural network. In the following, enriched GIPF descriptors and RDKit/Mordred descriptors were added to the model. They achieved an rmse 13.3 °C on their test set. While this approach is promising, it is important to note that the study focused solely on melting points in the range of 50–120 °C; thus, the applicability of their model is limited since the prediction outside this temperature range was not assessed. In their comparison to other benchmark models, other models achieved rmses between 14.0 and 15.1 °C on their data set. The most comprehensive model was established by Tetko et al.,^16^ which achieved a root mean squared error (rmse) below 33 °C for chemicals in the drug-like region (range of physicochemical properties possessed by marketed drugs). Based on this work, the authors even extended their model with data extracted from patents, including about 241,958 data points.^17^ Another approach was established by Yalkowsky and Alantary, where the MPs of 2,000 organic chemicals were calculated based on their structure, achieving an average absolute error of 38.6 °C.^1^

No approach with a data set in a broad temperature range reaches a prediction error lower than 30 °C, which is reasonable as it was determined that the experimental accuracy is in the range of 35 °C.^17^ Thus, we assume a limit for the prediction error at ∼35 °C due to the variance expected for the experimental MP data.

Artificial neural networks (ANNs) have become increasingly relevant in chemical research. They have already been applied in molecular design, synthesis planning, and toxicity prediction.^9,18^ The performance of the ANN models is highly dependent on the quality and quantity of the training data.^19^ A potential problem is that many data sets are imbalanced, while the algorithm assumes a well-balanced data set, which can affect parameter estimation.^20^ Furthermore, a big obstacle in science is that in many research fields, only small data sets are available, and it is very time-consuming to generate large data sets experimentally.^18^ To increase the data volume, data augmentation can be applied, which is a method to artificially increase the volume of the training data by modifying the corresponding data.^8^ T. Cirino used a data augmentation approach by the generation of tautomeric forms of molecules in the Tox24Challenge where the chemical binding to transthyretin should be predicted.^21^

In this study, we aim to predict MPs with a graph convolutional neural network (GNN) based on a data set of Bradley, which includes 28,645 MPs.^22^ We preprocessed the data set by excluding duplicates and marked faulty data points. We developed a GNN with an initial split of the data set into 70% training, 20% validation, and 10% test set. The corresponding test and validation set predictions were used to identify potential outliers in the initial data set, which were checked for decomposition and discrepancies in the experimental data points. Based on the curated data set, we improved the input data of the GNN, and therefore the corresponding performance. Further, we apply a data augmentation strategy based on generating different SMILES variants and tautomeric forms to enlarge the training set used to develop the GNN by a factor of ≈6. We evaluate our model’s performance based on the independent test set, which we split from the initial data set (10%), and compare our prediction results to an already existing model included in EPI Suite. We could demonstrate that the data augmentation strategy improves the performance of the GNN. For comparability with benchmark models, we applied our model on commonly used test sets.

## 2 Methods

### 2.1 Data Set and Data Curation

We obtained the MP data from Bradley’s MP data set, which consisted of 28,645 MPs, and we excluded duplicates to reduce distortion by frequently recorded compounds. The duplicates were identified based on the ChemSpider ID and the SMILES. Of the duplicates, one data point was randomly selected, and in total, 8,273 duplicates were sorted out. Further, 377 data points had the label “do not use” and were excluded.^22^ Frequently mentioned reasons for the label “do not use” were that the compounds are salts or mixtures or that the MP was out of range. The adjusted data set contained 19,995 data points. To obtain uniform SMILES in the data set, we converted them with OpenBabel 3.1.1. to canonical SMILES. We included a flowchart of the complete model development procedure in Figure 1.

**Figure 1:**
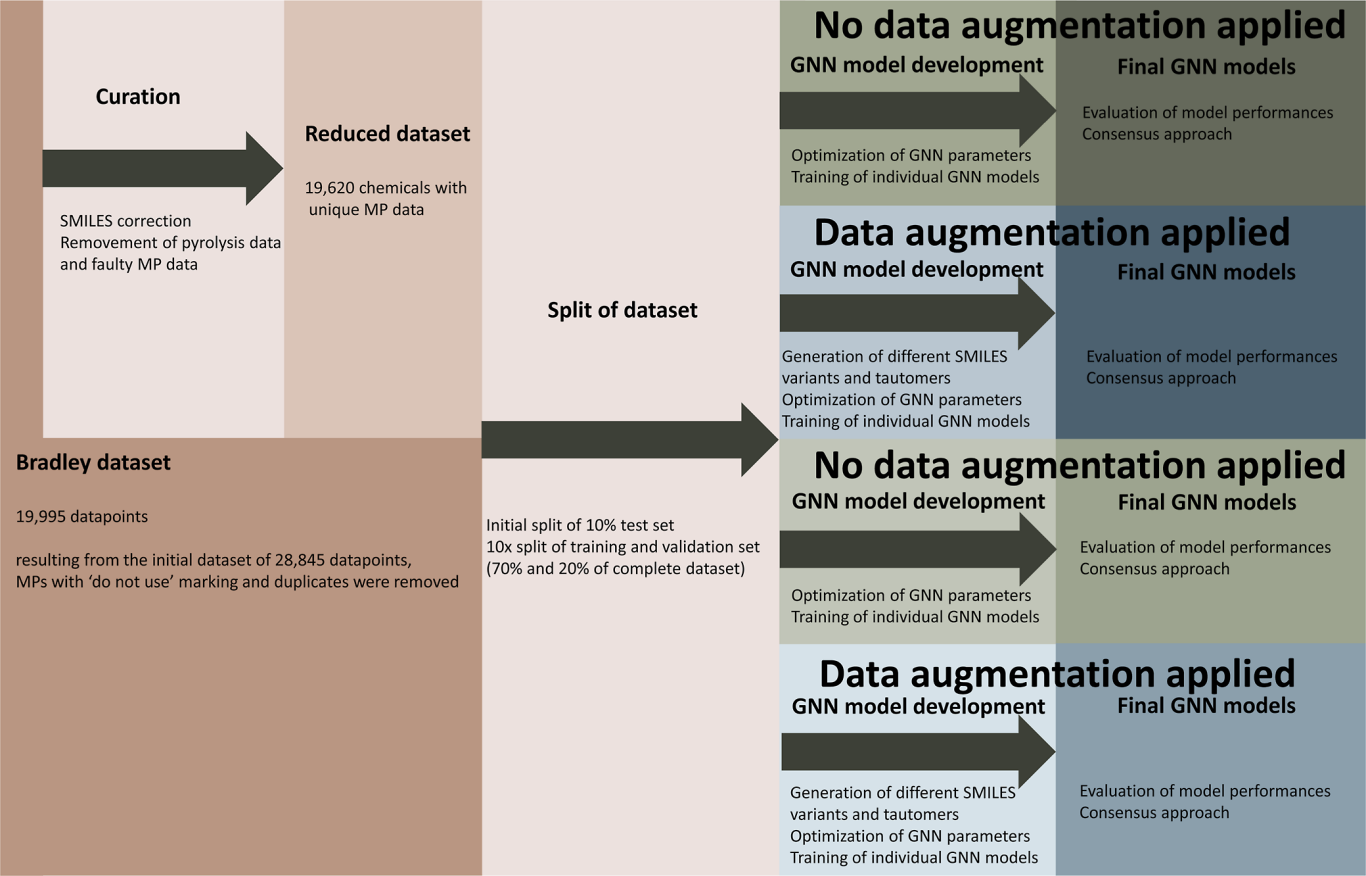
Flowchart to illustrate the data curation and model development.

We split the data set into ca. 70% training (13,989 data points), 20% validation (3,997 data points), and 10% test set (1,997) data points. After pretraining of some initial GNNs, we started with the identification of potential errors with significant influence on the performance because the used data set is not highly curated. Four splits were used to ensure that each MP was used at least once as either a validation or a test for an independent check. We used each split three times for the prediction. For data points where the predicted value deviated more than 80 °C from the experimental value in at least two predictions, we checked the value manually. In total, 1458 MPs were checked with different databases, including ChemSpider,^23^ Chemical Book,^24^ and SciFinder.^25^ When the MP in the corresponding databases deviated strongly from the MP in the data set, the MP was deleted if it was not clear which MP source is correct or if the compound decomposes instead of melting. If there was a clear data situation with several sources, the MP was adjusted. Further, the data set was compared with the decomposing compounds from the pyrolysis data set available at OChem and overlapping compounds were removed.^26^

Besides the cross-checks of MPs, the corresponding SMILES files were also checked. The main issue found was according to the given stereoisomeric information since the ChemSpider ID and the name suggested a stereochemistry, but the stereoinformation was not introduced into the SMILES. Thus, we performed an additional cross-check of the SMILES by extracting the corresponding information from the ChemSpider Web site based on their ChemSpider ID.^23^ Therefore, library ChemSpiPy and application programming interface keys were used. SMILES that could not be found with this extraction method were checked manually. In total, 2,077 SMILES were changed.

To allow for a cross-validation approach, we split the 10% for testing (randomly) from the curated data set and applied a 10-fold split on the remaining data set (details are provided in the data set included in the git repository).

### 2.2 Data Augmentation

For enlargement of the training set, we applied a data augmentation strategy. The reason for the data augmentation is that we want to use more neurons per layer without running into issues of overparameterization and to improve the prediction for underrepresented compounds. The original SMILES of the data set were maintained, and ten more SMILES variants were created with OpenBabel 3.1.1. The original SMILES were converted into canonical SMILES, inchified SMILES, and universal SMILES in the aromatic and the Kekulé form. Furthermore, the corresponding aromatic and Kekulé forms were created with explicit H’s for the canonical and universal SMILES. Afterward, the tautomers of the SMILES were generated using the open-source toolkit RDKit.^27^ Thereby, duplicates of SMILES were deleted. However, for SMILES codes with nitro groups in the trans position, no tautomers could be generated with RDKit. For these molecules, the stereo information was deleted to generate tautomers. Based on the possible tautomeric forms and the unique SMILES, the number of generated structures per molecule differs depending on the molecule. This is shown in the Supporting Information 1. If there were more than 50 different structures for one compound, then we randomly selected a maximum of 50 tautomeric forms/SMILES variants. Note that the same MP is assigned for each tautomeric form/SMILES variant. Based on this data augmentation approach, more SMILES are generated for larger and more complex molecules since they are more likely to have more tautomeric forms (Supporting Information 1). This can improve the prediction because the representation is reinforced for these molecules which are more difficult to predict and are underrepresented in the data set.

### 2.3 Development of the GNN

The GNN was developed in Python, version 3.11.8. Within Python, the libraries TensorFlow version 2.15.0, Keras version 2.15.0, and DeepChem version 2.7.2 ^28,29^ were used. The SMILES were transformed into molecular graphs with the ConvMolFeaturizer, which were then used as input for our GNNs. Our aim was to find a representation of local interactions and the global structure (regarding shape and size); thus, we selected the molecular graphs, where connectivities and chemical bonding are depicted. Here, atoms and their corresponding properties like the atom type, implicit valence, aromaticity, chirality, hybridization, and formal charge are represented as nodes, and the corresponding bonds to neighbor atoms are represented as edges. An aggregation of the features of neighbor atoms is done in the graph convolutional operation, which is depicted by a graph convolutional layer, followed by batch normalization, and graph pool layer. In total, we included two hidden layers; the output of the GNN includes a dense and a batch normalization. We applied a dropout of 0.1. We performed hyperparameter optimization based on the resulting plot of the corresponding rmses of the training and the validation set over the epochs. Different hyperparameters, like the number of neurons in the two hidden layers (including as well different forms like a pyramidal structure or the same number of neurons in both layers), learning rate, activation function, and loss function, were varied. We, therefore, started with different setups of the neural networks, including various numbers of neurons per layer and varied the corresponding learning rates starting from 0.00001 up to 0.1. We further tested different loss functions and activation functions in various combinations for the best setups, regarding the number of neurons per hidden layer. For model optimization, an overall number of 200 epochs was used for each combination of hyperparameters. Details can be found in Supporting Information 2 and 3. For the training of the model without data augmentation, the hidden layers had 32 and 64 neurons; for the training with data augmentation, we increased the number of neurons to 64 and 128 (Supporting Information 4). We applied early stopping in our final trained models with a patience of 20. The model training was conducted on the EVE cluster, a high-performance computing cluster operated by the Helmholtz Center for Environmental Research (UFZ).

## 3 Results and Discussion

### 3.1 Data Set used for Model Generation

The data set of Bradley^22^ was randomly split into training set (70%), validation set (20%), and test set (10%). In brief, we split the test set first and applied a 10-fold random split for the remaining set. Thus, we ended up with ten different variants of splits, allowing for a cross-validation approach (details are provided in the data set included in the git repository). In Figure 2A, the distribution of the MPs of the reduced Bradley data set^22^ is shown. The MPs range from −205 to 517 °C. In total, 69% of the MPs are in the range of 50–250 °C, which is the drug-like region.^17^ This interval is especially interesting for pharmaceutical and toxicological research. The drug-like region is marked in Figure 2A with the dashed lines. Below the drug-like region, the data density increases strongly for MPs between 0 and 50 °C. In the drug-like region, compounds with MPs of 50 to 170 °C are well represented, but with further increasing MPs, the data density decreases. The diagram shows that the data set is not balanced, which can lead to lower accuracy in predicting underrepresented MPs, such as low or high MPs.

**Figure 2:**
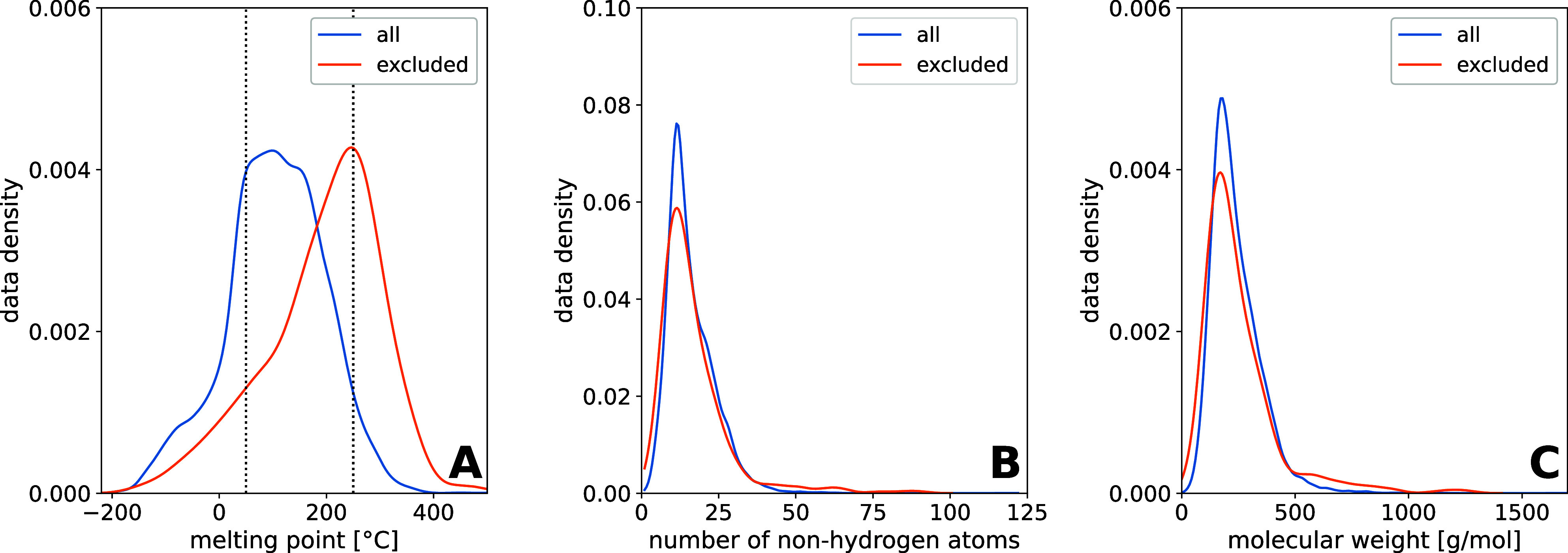
Comparison of the data density of the reduced Bradley data set (19,995 data points) with the data density of the excluded data points (367 data points), which we identified as errors, as a function of the MP (A), as a function of the number of non-hydrogen atoms (NHA) (B), and as a function of the molecular weight (C). Note that the data density is normalized according to the data set depicted (in the case of the errors, the data set including all data points identified as errors, is the reference).

Additionally, the data density according to the number of NHA is shown in Figure 2B. Therein, it can be seen that the data density increases drastically from 1 to 12 NHA and is the highest for 12 NHA. The data density in this area and the structures are relatively simple; therefore, there is a lot of experimental data available for these compounds. For a number of non-hydrogen atoms (NHA) higher than 12, the data density decreases again until 40. Molecules with a number of NHA higher than 40 are scarce. Molecules with high numbers of NHA are often complex in structure and synthesis; thus, the availability of experimental data is limited. Since the MP increases with increasing molar mass, it is expected to increase also with increasing NHA. However, it must be considered that all NHA are regarded with the same weight. At the same time, the different atoms have different molar masses and thereby have different influences on the total molar mass of the molecule. The data density as a function of molar mass is shown in Figure 2C.

### 3.2 Data Curation Procedure and Data Density of Errors Identified in the Data Set

According to our data curation workflow (depicted in Figure 1), we excluded 367 compounds and corrected the MPs of 49 compounds. By individual literature review of data points with a strong deviation in the prediction, 271 decomposing chemicals and chemicals with strongly deviating data sources for MPs were sorted out. Additionally, 96 chemicals were identified, where decomposition occurred by comparison to a corresponding data set.^17^ In Figure 2, we depicted the data density of the errors identified in the data set compared to the data density of the complete data set. The data density is normalized according to the data set depicted (in the case of the identified errors, the corresponding error data set is the reference).

When looking at the data density as a function of the MP, it can be seen that at a higher temperature, MP data errors occur more frequently, and the data density of the complete data set is low at this region. The majority of excluded data points (283 data points) are compounds that decompose instead of melting. Since decomposition of chemicals is expected to occur more often in the range of higher temperatures, this could be an explanation for the observed shift of the densities of the errors to higher MPs. Further, the data density of the errors as a function of the MP is slightly shifted to a higher number of NHAs and higher molar masses (Figure 2B,C). This can be seen for numbers of NHA >40 and molar masses >500 g/mol in the corresponding graphs.

### 3.3 Influence of Data Augmentation on the Data Density

Data augmentation is a method to enlarge the data set. We applied data augmentation to our training sets by generating different variants of SMILES and different tautomers. Since the number of unique SMILES and tautomers is not the same for every molecule, the data augmentation leads to a shift in the data density. In Figure 3, the influence of data augmentation on the data density as a function of the MP (A), the number of NHA (B), and the molecular weight (C) is shown, using an exemplary split of the training set. It can be seen that the data density is shifted to higher melting points. Furthermore, the data density is shifted to higher numbers of NHA and higher molecular weights. This can be explained by the fact that larger molecules tend to have more possible unique SMILES and tautomeric forms.

**Figure 3:**
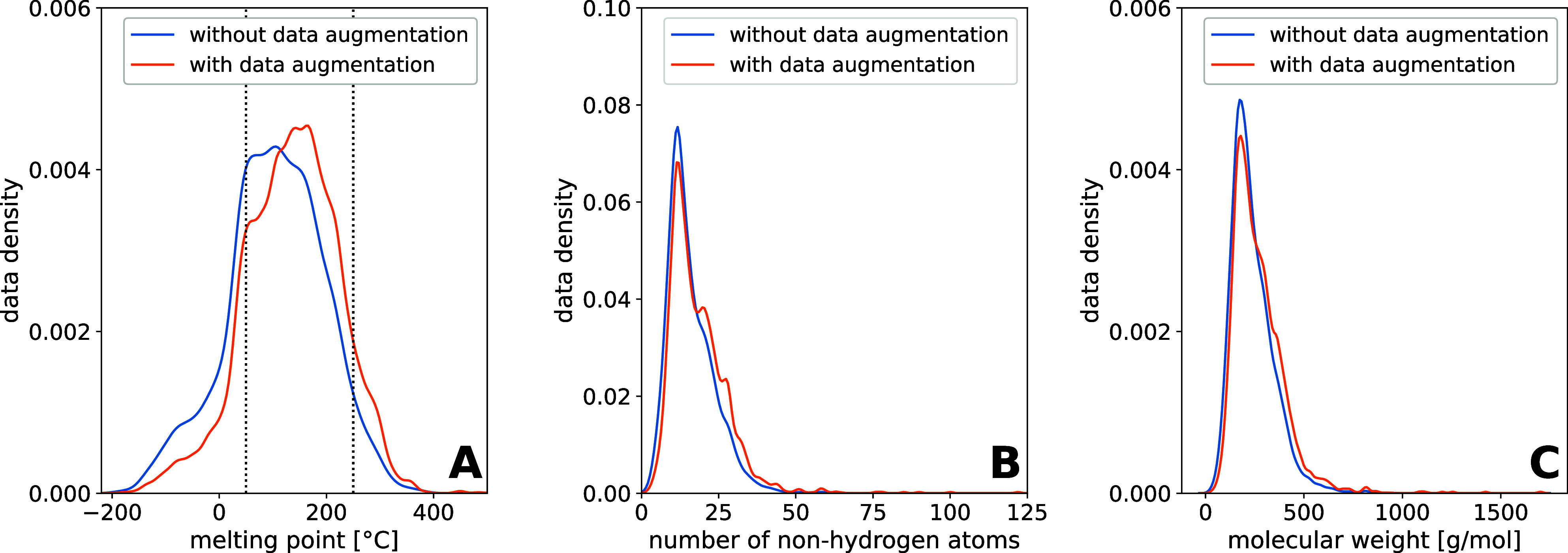
Comparison of the data density of an exemplary split of the training set without (13,989 data points) and with data augmentation applied (86,081 data points), as a function of the MP (A), as a function of the number of NHA (B), and as a function of the molecular weight (C). Note that the data density is normalized according to the data set depicted.

### 3.4 Model Development and Performance

To find the optimal parameter setup for our GNNs, models were trained over 200 epochs, and the rmse of the training and validation set was plotted versus the number of epochs. The number of neurons in the hidden layers as well as the learning rate, the activation function, the batch size, and the dropout layer were varied. The details can be found in Supporting Information 2 and 3.

Based on the initial and the curated data set, we developed two different GNN modeling approaches in each case, one where we did not apply data augmentation (∼14,000 training set input features) and one where we applied a data augmentation strategy based on the generation of tautomeric forms and different SMILES variants (∼85,000 training set input features). The GNN models, where we did not apply data augmentation, include two layers with 32 and 64 neurons. Leaky ReLU activation functions, a learning rate of 0.0005, and an L1Loss function were applied. We used early stopping during the training. The GNN, where we applied data augmentation, was also trained using the early stopping approach, with a structure of two hidden layers (64 and 128), a learning rate of 0.0005, a leaky ReLU activation function, and an L1Loss function. We used the independent test set of 1,961 chemicals (reduced after data curation) to evaluate the performance of both models. Note that we assign the same MPs (experimentally observed MP) to all tautomeric forms and SMILES variants. In reality, the MP results from the sum of tautomer-specific MPs based on the abundance of the tautomer. The advantage of our method is that the macroscopic value (observed MP for the corresponding chemical) will be predicted regardless of which tautomeric form is queried.

We applied a 10-fold split of training and validation set in each model variant trained, allowing for a cross-validation. The results are shown in Table 1.

**Table 1:** Results of the 10-fold Cross-Validation for the Four Different Model Types Developed, Namely, the Models Developed on the Initial Dataset and the Models Developed on the Curated Dataset, Where We did Use the Dataset Itself for Training and Where We Applied a Data Augmentation Strategy

	initial data set						curated data set					
	validation set			test set^b^			validation set			test set		
model	*r* ^2^	*q* ^2^	rmse^a^	*r* ^2^	*q* ^2^	rmse^a^	*r* ^2^	*q* ^2^	rmse^a^	*r* ^2^	*q* ^2^	rmse^a^
GNN1	0.784	0.737	42.9	0.773	0.726	42.8	0.782	0.740	41.7	0.772	0.731	42.9
GNN2	0.783	0.747	43.1	0.780	0.752	42.1	0.801	0.753	40.8	0.782	0.738	42.0
GNN3	0.787	0.755	42.3	0.796	0.772	40.6	0.800	0.756	41.0	0.783	0.735	41.9
GNN4	0.792	0.756	42.4	0.785	0.754	41.7	0.782	0.748	42.3	0.783	0.752	41.9
GNN5	0.797	0.758	41.3	0.767	0.729	43.4	0.801	0.759	41.4	0.777	0.736	42.5
GNN6	0.767	0.737	43.6	0.785	0.761	41.7	0.810	0.778	40.7	0.783	0.749	41.8
GNN7	0.781	0.744	42.7	0.781	0.745	42.0	0.781	0.720	43.5	0.770	0.709	43.1
GNN8	0.766	0.717	45.2	0.768	0.730	43.3	0.801	0.776	39.9	0.774	0.740	42.7
GNN9	0.785	0.756	42.2	0.764	0.735	43.7	0.786	0.742	42.2	0.788	0.731	41.4
GNN10	0.789	0.733	43.2	0.770	0.730	43.1	0.811	0.793	40.4	0.782	0.760	41.9
average	0.783	0.744	42.9	0.777	0.743	42.4	0.795	0.756	41.4	0.780	0.738	42.2
SD	0.009	0.013	1.0	0.009	0.015	0.9	0.011	0.020	1.0	0.005	0.013	0.5

	initial data set, data augmentation applied						curated data set, data augmentation applied					
	validation set			test set^b^			validation set			test set		
model	*r* ^2^	*q* ^2^	rmse^a^	*r* ^2^	*q* ^2^	rmse^a^	*r* ^2^	*q* ^2^	rmse^a^	*r* ^2^	*q* ^2^	rmse^a^
GNN1	0.815	0.775	39.7	0.804	0.760	39.8	0.808	0.771	39.1	0.820	0.784	38.1
GNN2	0.809	0.771	40.5	0.817	0.781	38.5	0.830	0.793	37.7	0.821	0.789	38.1
GNN3	0.812	0.772	39.8	0.814	0.776	38.7	0.823	0.784	38.6	0.810	0.769	39.2
GNN4	0.813	0.773	40.2	0.811	0.777	39.1	0.809	0.761	39.5	0.805	0.754	39.7
GNN5	0.819	0.791	39.0	0.806	0.777	39.6	0.822	0.775	39.1	0.815	0.773	38.7
GNN6	0.787	0.742	41.7	0.811	0.777	39.1	0.824	0.788	39.1	0.818	0.780	38.4
GNN7	0.811	0.764	39.7	0.805	0.761	39.7	0.816	0.774	39.8	0.810	0.770	39.2
GNN8	0.807	0.761	41.1	0.816	0.774	38.6	0.825	0.800	37.4	0.812	0.780	39.0
GNN9	0.814	0.775	39.3	0.808	0.773	39.4	0.813	0.777	39.5	0.804	0.764	39.8
GNN10	0.817	0.769	40.2	0.816	0.775	38.6	0.832	0.801	38.1	0.821	0.788	38.0
average	0.810	0.769	40.1	0.811	0.773	39.1	0.820	0.782	38.8	0.814	0.775	38.8
SD	0.009	0.012	0.8	0.005	0.007	0.5	0.008	0.012	0.8	0.006	0.011	0.6

a The rmse is given in °C.

b Note that we used the test set of the curated data set to allow for a direct comparison.

In general, it can be seen from the results of the cross-validation (Table 1), that the values for *r* ^2^ and *q* ^2^, and the rmse for the four different variants of GNNs are close to each other for the ten splits and the different model variants.

The corresponding *r* ^2^ values are close to each other for each of the model variants and the *q* ^2^ values are close to the corresponding *r* ^2^ values. The rmse values for the models, where no data augmentation was applied for training are in the range of 40.6–43.7 °C for the models developed on the initial data set and 41.4–43.1 °C for the models developed on the curated data set. For the two model variants, where data augmentation was applied, the rmse values were in the range of 38.5–39.8 °C (initial data set) and 38.0–39.8 °C (curated data set). The average rmse values are slightly lower for the models developed on the curated data set, but no significant difference is observed.

Based on the 10-fold splits, we developed four different consensus GNNs. The evaluation of the models is depicted in Table 2.

**Table 2:** Performance of the Four Different Consensus GNNs Developed on the Initial Dataset and Curated Dataset for Models, Where Data Augmentation Was Applied and Models, Where No Data Augmentation Was Applied

	no data augmentation applied		data augmentation applied	
	initial data set	curated data set	initial data set	curated data set
rmse	37.8 °C	38.0 °C	35.4 °C	35.4 °C
*r* ^2^	0.824	0.823	0.847	0.847
*q* ^2^	0.787	0.777	0.806	0.806
mne	–260.7 °C	–246.6 °C	–175.0 °C	–175.7 °C
mpe	244.0 °C	226.9 °C	220.4 °C	209.4 °C
bias	2.3	3.3	3.5	2.8

The consensus GNNs, where no data augmentation was applied, are relatively close to each other with *r* ^2^ values of 0.824 and 0.823 for the models developed on the initial data set and the curated data set, respectively. The corresponding *q* ^2^ values are close to the *r* ^2^ values (0.787 for the models developed on the initial data set and 0.777 for the models developed on the curated data set). The rmse for the model developed on the initial data set is slightly lower (37.8 °C) than for the consensus GNN developed on the curated data set (38.0 °C). The bias for the consensus GNN developed on the initial data set is also lower compared to the bias of the consensus GNN developed on the curated data set. However, the maximum negative error (MNE) and maximum positive error (MPE) are lower for the consensus GNN developed on the curated data set, which could be explained by the curation itself.

For the GNNs, where data augmentation was applied, the situation is slightly different. Here, the consensus GNN achieves for both models an rmse of 35.4 °C. The *r* ^2^ and *q* ^2^ values are the same for both variants of the consensus GNNs. The maximum negative error observed is slightly higher for the consensus GNN developed on the curated data set (−175.7 °C vs −175.0 °C for the consensus GNN developed on the initial data set). For the maximum positive error, the value is lower for the consensus model developed on the curated data set (209.4 °C vs 220.4 °C for the consensus GNN developed on the initial data set). The bias of the corresponding model is also lower with a value of 2.8, the bias for the model developed on the initial data set is 3.5. The rmse values for the data augmentation approaches are significantly lower, which is, from our perspective, an argument for the application of this approach.

The differences in the model performances based on the training with the two different data sets are small and indicate that the curation does not improve the prediction performance. Thus, the curation is not needed for the development of the model based on the used data set.

We additionally tested whether the structure of the GNN itself, which is different for both variants (with and without data augmentation), has an impact on the different performance outcomes. We therefore developed a consensus GNN without the application of our data augmentation strategy using the curated data set with a setup including 64 and 128 neurons (as used for the models where we did apply data augmentation). The corresponding rmse value of the consensus GNN was 37.5 °C, slightly better compared to the consensus GNN trained by using 32 and 64 neurons. However, overparameterization occurred in this case (more details are provided in Supporting Information 5).

We further investigated the corresponding standard deviations (SDs) of the test set predictions of the ten models applied for each model variant (Table 3). We grouped the SDs and inserted quality and reliability criteria for each group. For SDs in the range of 0 to 10 °C, we defined a high quality and reliability. This is observed for 301 chemicals of the test set for the model variant, where we used the initial data set for training and did not apply data augmentation. The number is increased to 336 chemicals for the corresponding model variant developed on the curated data set. The rmse values are low with 29.7 and 23.7 °C, respectively. If data augmentation is applied, the number of chemicals in this group is higher with 364 chemicals (for the initial data set model variant, rmse 24.3 °C) and 424 chemicals (for the variant trained on the curated data set, rmse 26.7 °C).

**Table 3:** Overview of the Number *n* of Chemicals and the Corresponding rmse Values for the Four Different Consensus GNNs Developed in the Corresponding SD Range

		no data augmentation applied				data augmentation applied			
SD range		initial data set		curated data set		initial data set		curated data set	
[°C]	quality/reliability criteria	*n*	rmse [°C]	*n*	rmse [°C]	*n*	rmse [°C]	*n*	rmse [°C]
0–10	high quality/reliability	301	29.7	336	23.7	364	24.3	424	26.7
10–20	good quality/reliability	1,155	33.5	1,135	35.1	1254	33.7	1,209	33.9
20–30	moderate quality/reliability	383	42.9	371	46.7	293	47.3	283	45.4
>30	low quality/reliability	122	66.4	119	60.3	50	58.7	45	64.0

We assigned a good quality and reliability for SDs in the range of 10 to 20 °C. In total, 1,155 chemicals are included in this group for the model variant trained on the initial data set without data augmentation, the rmse value is 33.5 °C for this group. 1,135 chemicals are included in the corresponding group for the model variant developed on the curated data set; the rmse is 35.1 °C. For the models, where data augmentation was applied, 1,254 chemicals (initial data set) and 1,209 chemicals (curated data set) are included in this group. The corresponding rmse values are 33.7 and 33.9 °C, respectively.

The group with SDs ranging from 20 to 30 °C includes 383 test set chemicals (initial data set) and 371 chemicals (curated data set) for the model variants developed without data augmentation. The corresponding rmse values are 42.9 and 46.7 °C, respectively. For the model variants developed by the use of a data augmentation strategy, 293 chemicals (initial data set for training) and 283 chemicals (curated data set for training) are included in this group. The rmse values are 47.3 and 45.4 °C, respectively.

In the group of SDs greater than 30 °C, 122 chemicals (initial data set, rmse 66.4 °C) and 119 chemicals (curated data set, rmse 60.3) are included for the model variants where no data augmentation was applied. For the model variants, where data augmentation was applied, 50 chemicals (initial data set, rmse 58.7 °C) and 45 chemicals (curated data set, rmse 64.0 °C) were included.

As can be seen from Table 3, the number of chemicals in the low quality group is drastically reduced by the application of data augmentation for training the model variants. Further, the number of chemicals in the high quality group and good quality group is increased as well. However, data quality (initial versus curated data set) does not have a strong impact. Thus, data augmentation has a substantial impact on the performance of the model variants.

### 3.5 Performance Dependency on the Different MP Ranges

Since our data set is imbalanced regarding the temperature, it can be expected that the model performs differently depending on the temperature ranges. The rmses for three different temperature regions are given in Table 4. A region of special interest is the drug-like region (50–250 °C). In this region, the model without data augmentation reaches an rmse of 36.9 and 37.0 °C when trained on the initial and the curated, respectively. The performance is increased for the GNN model with data augmentation to an rmse of 34.8 and 34.7 °C, respectively. Below the drug-like region, the performance of both models is even better. The GNN without data augmentation reaches an rmse of 32.7 °C when trained on the initial and an rmse of 32.3 °C when trained on the curated data set. The GNN model with data augmentation achieves an rmse of 30.6 °C for the initial data set and 31.4 °C for the curated data set. Above the drug-like region, the performance is drastically decreased. Without data augmentation, the model reaches an rmse of 71.2 °C when trained on the initial data set and an rmse of 74.1 °C when trained on the curated data set. For the model with data augmentation, the rmse is improved for both the initial and the curated data set (64.9 and 62.9 °C). The poor prediction performance above the drug-like region can be explained by the low data density in this region, as shown in Figure 2A. This is also represented in the small number of compounds in the test set with 76.

**Table 4:** Temperature-Dependent Prediction Performance for the Different Models for the Test Set: Comparison of the GNN Model without and with Application of Data Augmentation Trained with the Initial and Curated Dataset and the Prediction Performance of the EPI Suite Tool^a^

	GNN					EPI suite	
		no data augmentation applied		data augmentation applied			
		initial data set	curated data set	initial data set	curated data set		
region of prediction	*n*	rmse [°C]	rmse [°C]	rmse [°C]	rmse [°C]	*n*	rmse [°C]
complete data set	1,961	37.8	38.0	35.4	35.4	1,955	62.0
below 50 °C	505	32.7	32.3	30.6	31.4	503	57.3
drug-like region (50–250 °C)	1,380	36.9	37.0	34.8	34.7	1,376	61.2
above 250 °C	76	71.2	74.1	64.9	62.9	76	75.7

a By the use of EPI Suite, not all MPs of the test set chemicals could be predicted. Thus the number of test set chemicals is lower in this case.

Additionally, we used the EPI Suite (EPIWEB version 4.1) to predict the MPs for the chemicals of our test set. Here, we only used predicted values to allow for a direct comparison of the models. The results for the predictions can be found in Table 4 and Figure 4B. As can be seen here, there is an explicit cutoff for the maximum predicted MPs at ≈350 °C, indicating a limitation of the EPI Suite to that maximum MP temperature. The rmse of the predictions performed by EPI Suite for the test set chemicals is much higher (rmse = 62.0 °C, Table 1). As can be seen from Figure 4B, there are more outliers observed with greater differences between the experimental and predicted MP (ΔMP >100 °C) as compared to the predictions of the GNN model trained on the curated data set with data augmentation­(Figure 4A, plots of the three other consensus GNNs are shown in Supporting Information 6).

**Figure 4:**
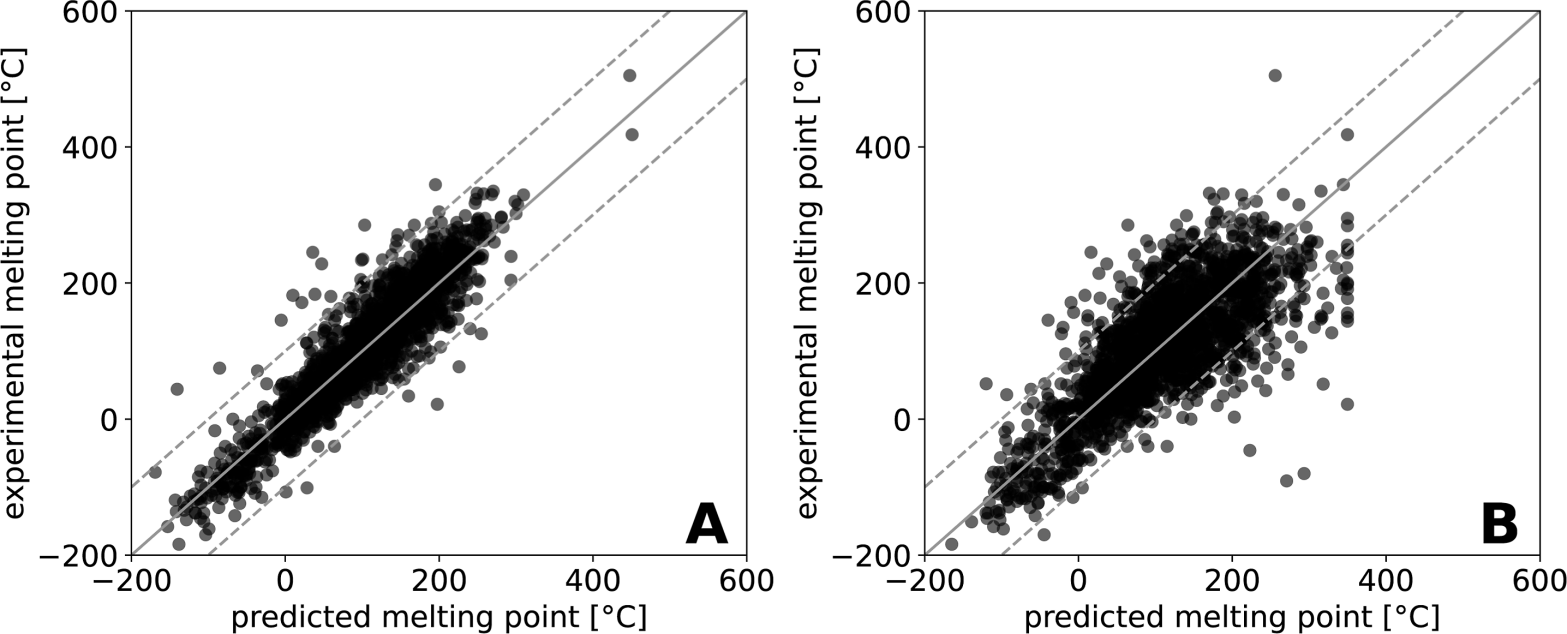
Experimentally determined MPs are plotted against the predicted ones for the model trained on the curated data set with data augmentation (A) and the MP prediction tool of EPI Suite (B).

### 3.6 Performance Dependency on the Number of Non-Hydrogen Atoms

As in our data augmentation approach, more structures are generated for larger and more complex molecules, leading to a shift of the data density (Figure 5A,B). The influence of the shift to larger molecules is assessed in Figure 5 for models trained with the initial data set (Figure 5A,C) and the curated data set (Figure 5B,D). For both data sets, it can be seen that the model without data augmentation gives more strongly deviating predictions, especially for larger molecules than the model with data augmentation (Figure 5A,B). Figure 5C,D shows that from the interval of 20–30 molecules, the performance of the model improves with data augmentation. The strong increase of the rmse at high numbers of NHA can be attributed to the low data density in this region, which is visible in Figure 5A,B.

**Figure 5:**
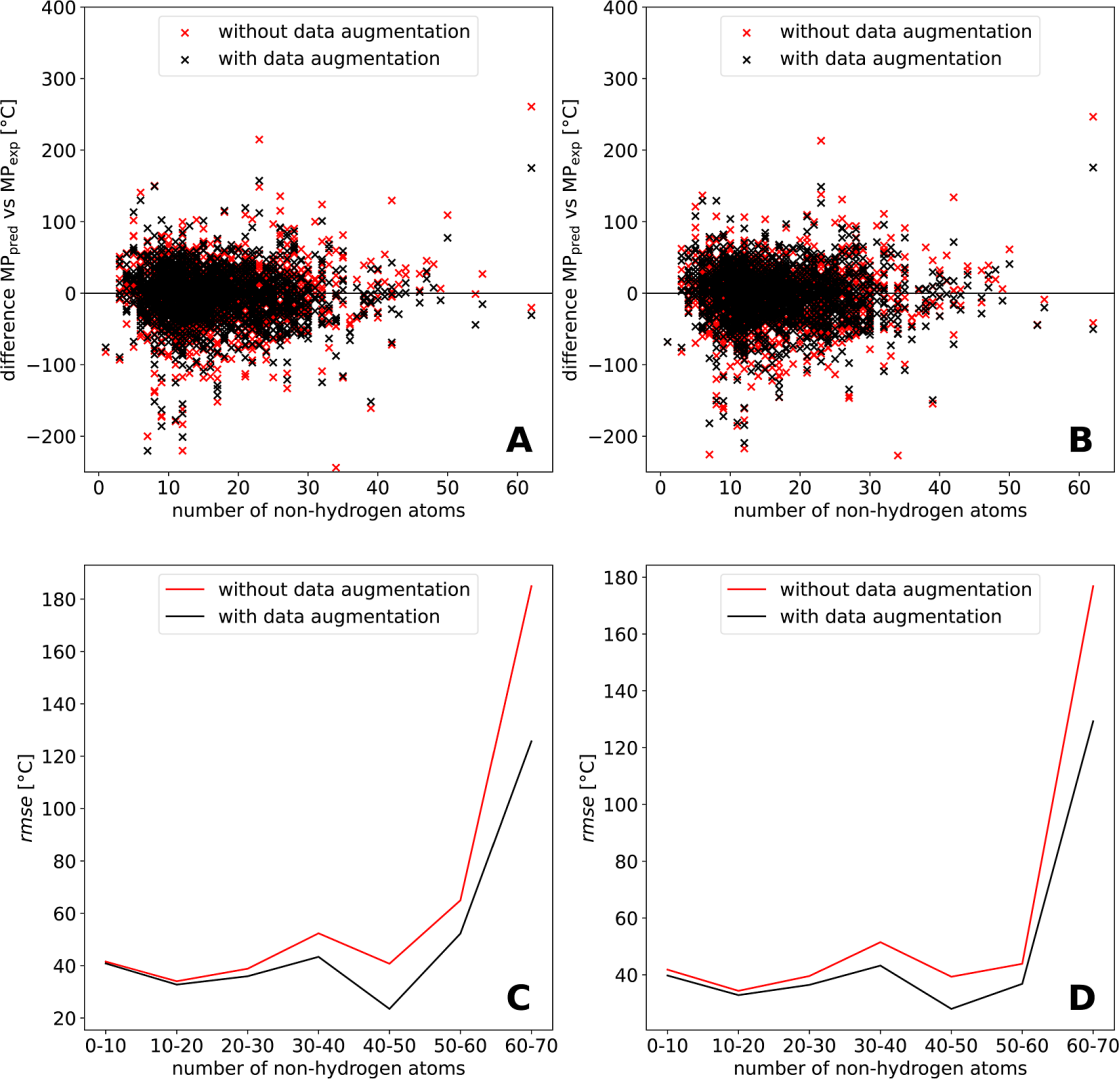
Difference between the experimental and predicted MPs is plotted versus the number of NHA for the test set chemicals trained with the initial data set (A) and the curated data set (B) for the models without data augmentation and with data augmentation applied. The rmse was calculated for the intervals of 10 NHA and is plotted as function of the intervals of NHA for the models trained on the initial data set (C) and the curated data set (D).

We further selected three different data sets from literature to test and to compare the performance of our developed consensus GNN to established models from literature (Table 5),^16^ namely, the Bradley set (initial smaller data set of 2,886 data),^22^ the Bergström data set (277 chemicals),^30^ and the Enamine data set (22,404 chemicals).^31^ For the Bradley data set in this publication, highly curated data points were used, including only MPs where at least two sources were available and which do not differ more than 5 °C. Our consensus GNN model (based on the curated data set, applying data augmentation) achieved an rmse of 19.0 °C on the Bradley set, which might be explained by the fact that 2,604 of the chemicals in the data set are included in our training and validation data sets. Thus, we removed the corresponding chemicals in our training set and validation set and repeated splits and the training of the ten GNN models to come up with an consensus model, which achieves an rmse of 32.4 °C. Tetko et al. achieved an rmse of 33.9 °C on the Bradley set, as they applied all developed MP models (including one which was established on this data set) as a consensus approach for the prediction of the data set.^16^

**Table 5:** Prediction Outcomes of the Consensus GNN (with Data Augmentation) for Three Different Test Sets from Literature

		trained on the curated data set	trained on the curated data set with removed duplicated
data set	*n* test set	rmse [°C]	rmse [°C]
Bergström	277	17.7	36.9
Bradley	2,886	19.0	32.4
Enamine	22,404	41.5	41.5

For the data set of Bergström et al.,^16,30^ our model achieved an rmse of 17.7 °C. Again, the very low rmse value can be explained by the chemical structures in the data set, which are covered by our training set. When removing 224 overlapping chemicals from our training set and validation set used and the corresponding Bergström data set, a performance of 36.9 °C was achieved (by retraining of the consensus GNN). The consensus model of Tetko et al. showed an rmse of 34.0 °C.

As a third data set, we predicted the MPs for the chemicals of the Enamine data set. Here, we could achieve a rmse of 41.5 °C. As there was only little overlap of 50 from 22,404 data points between the training and validation data set and the Enamine data set, the rmse did not change by removing the overlapping chemicals. Compared to our performance, the rmse of the consensus model of Tetko et al. was lower with an rmse of 39.8 °C.^16^

## 4 Conclusions

We investigated how data curation and data augmentation impact the performance of GNNs for the prediction of MPs. We found that data curation has almost no impact on the prediction performance. This might be different for smaller data sets. However, by applying a data augmentation strategy to enlarge the number of input features, we could increase the overall predictive performance. The increased number of input features allows for an extension of the structure of the GNN with respect to the neurons included per hidden layer, which leads to a higher resolution without problems occurring due to overparametrization. Our model provides accurate prediction results with an rmse of 35.4 °C on the independent test set. The performance of the consensus GNN might be limited by the experimental variance. The error for the experimental determination of the MPs is approximately 35 °C. Lower rmses can only be achieved by modeling on higher curated data sets with a small experimental variance. These are restricted to a limited variety in chemical structures, making the model less generalizable and leading to a smaller application domain. We will implement our models in our software PAULY. Furthermore, we want to implement a classification model to determine whether a compound decomposes.

## Supplementary Material



## Data Availability

The underlying data sets and python code for this study are available in the GIT repository nadinulrich/MP_prediction: https://github.com/nadinulrich/MP_prediction.

## Notes

Distribution of the SMILES variants and tautomers generated and dependence on the molecular weight; hyperparameter optimization of the GNN models for training without data augmentation applied; hyperparameter optimization of the GNN models for training with data augmentation applied; hyperparameter setup for the final nets including a comparison of the differences regarding the topology; evaluation of the impact of the topology applied in the different GNN models on the overall performance; and prediction performance of the different consensus GNNs (PDF)
