# Supplementary material for: Prediction of Melting Points of Chemicals with a Data Augmentation-Based Neural Network Approach
Source: ACS Omega. 2025 Jun 3;10(23):24296–306. doi: 10.1021/acsomega.5c00205 (PMC12177604; doi:10.1021/acsomega.5c00205)
Supplement: Supplementary file 1 [file ao5c00205_si_001.pdf]

Supplementary Information - Prediction of melting points of chemicals with a data augmentation-based neural network approach

Lea E. Austermeier[1], Karsten Voigt[2], Alexander Böhme[1], Nadin Ulrich[1,2]*

[1]Department of Exposure Science, Helmholtz Centre for Environmental Research – UFZ, Permoserstrasse 15, D-04318 Leipzig, Germany

[2]PAULY, Theresienstrasse 50, D-04129 Leipzig, Germany

*Corresponding Author: Department of Exposure Science, Helmholtz Centre for Environmental Research – UFZ, Permoserstrasse 15, D-04318 Leipzig, Germany

E-mail: nadin.ulrich@ufz.de

Table of content

SI1 Distribution of the SMILES variants and tautomers generated and dependence on the molecular weight. 2

SI2 Hyperparameter optimization of the GNN models for training without data augmentation applied. 3

SI3 Hyperparameter optimization of the GNN models for training with data augmentation applied. 6

SI4 Hyperparameter setup for the final nets including a comparison of the differences regarding the topology. 9

SI5 Evaluation of the impact of the topology applied in the different GNN models on the overall performance 10

SI6 Prediction performance of the different consensus GNNs. 10

SI1 Distribution of the SMILES variants and tautomers generated and dependence on the molecular weight.

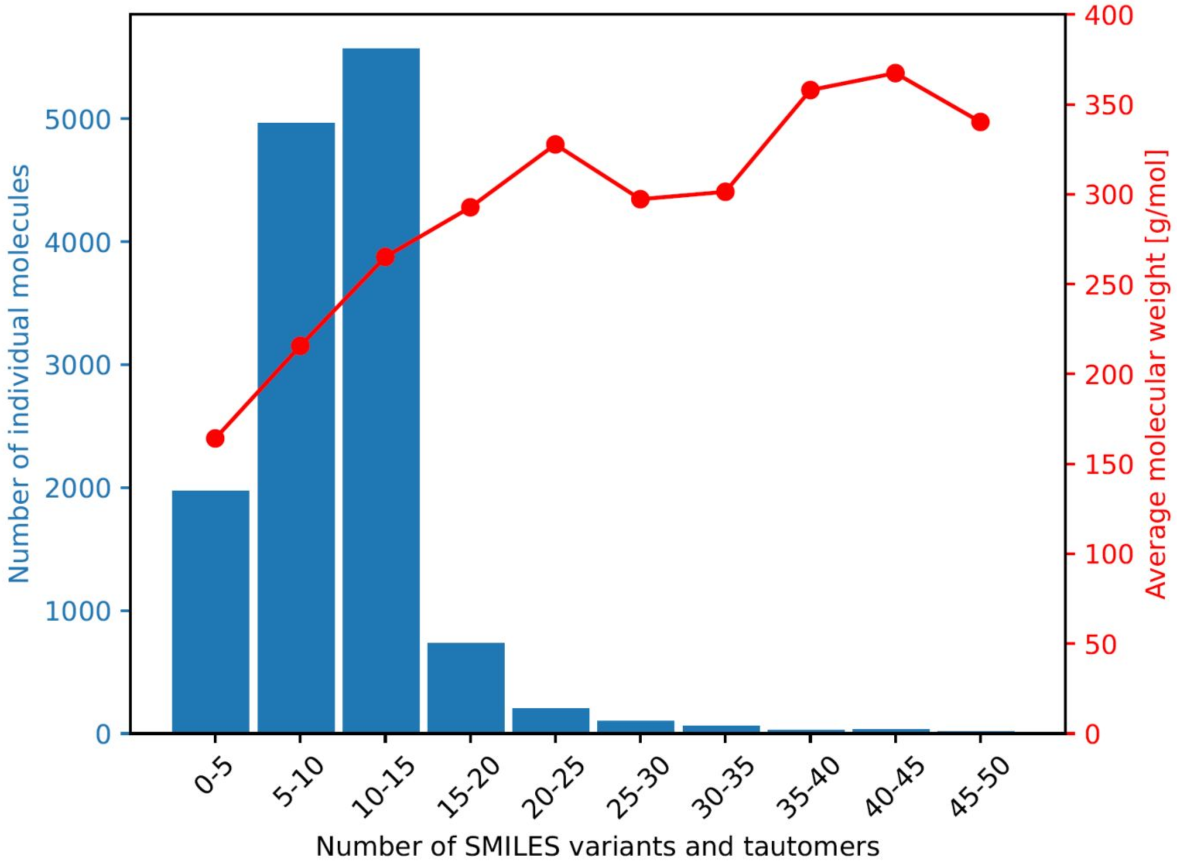

Figure S1. Histogram of the number of SMILES variants and tautomers generated in the data augmentation process (blue). Average molecular weight as function of the intervals of number of SMILES variants and tautomers.

SI2 Hyperparameter optimization of the GNN models for training without data augmentation applied.

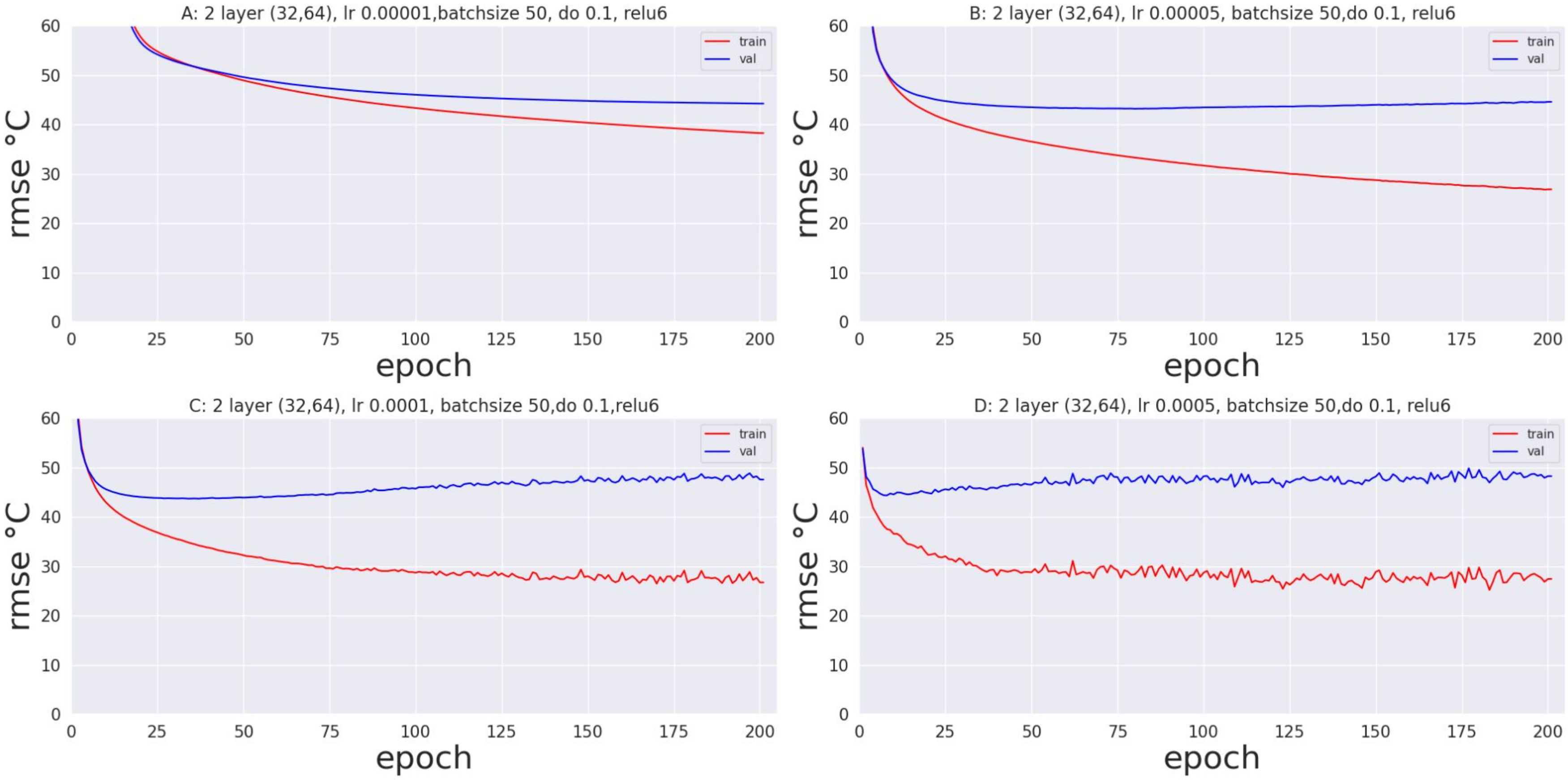

Figure S2. Plots of the *rmse* over the epochs to select the optimal parameters for the training of the neural networks for training set (red) and validation set (blue). Two hidden layers with 32 and 64 neurons are included, a ReLU6 activation function is applied, the dropout is set to 0.1 and a batch size of 50 is used. The learning rate (lr) is varied: (A) lr=0.00001, (B) lr=0.00005, (C) lr=0.0001, and (D) lr=0.0005.

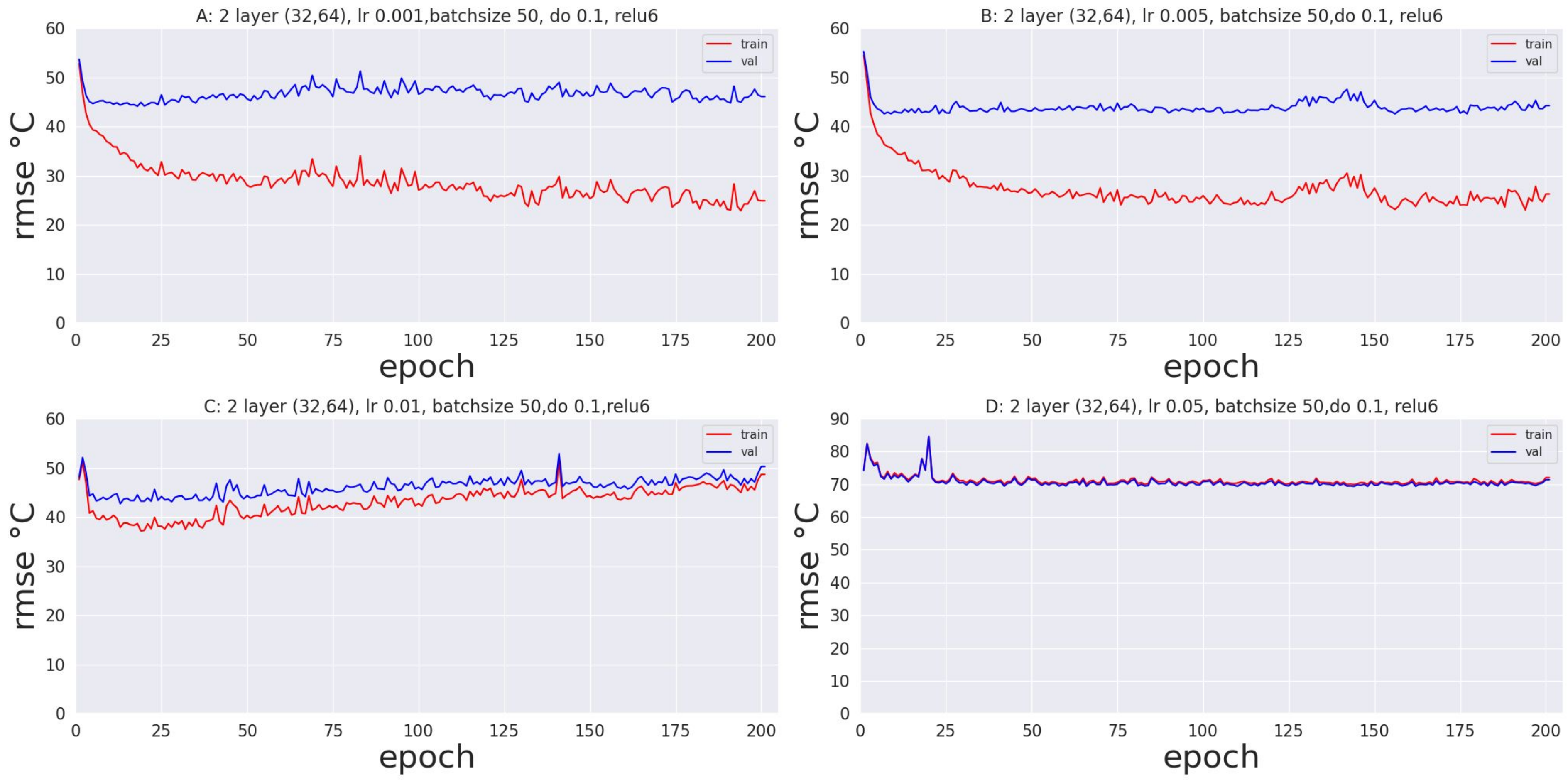

Figure S3. Plots of the *rmse* over the epochs to select the optimal parameters for the training of the neural networks for training set (red) and validation set (blue). Two hidden layers with 32 and 64 neurons are included, a ReLU6 activation function is applied, the dropout is set to 0.1 and a batch size of 50 is used. The learning rate (lr) is varied: (A) lr=0.001, (B) lr=0.005, (C) lr=0.01, and (D) lr=0.05.

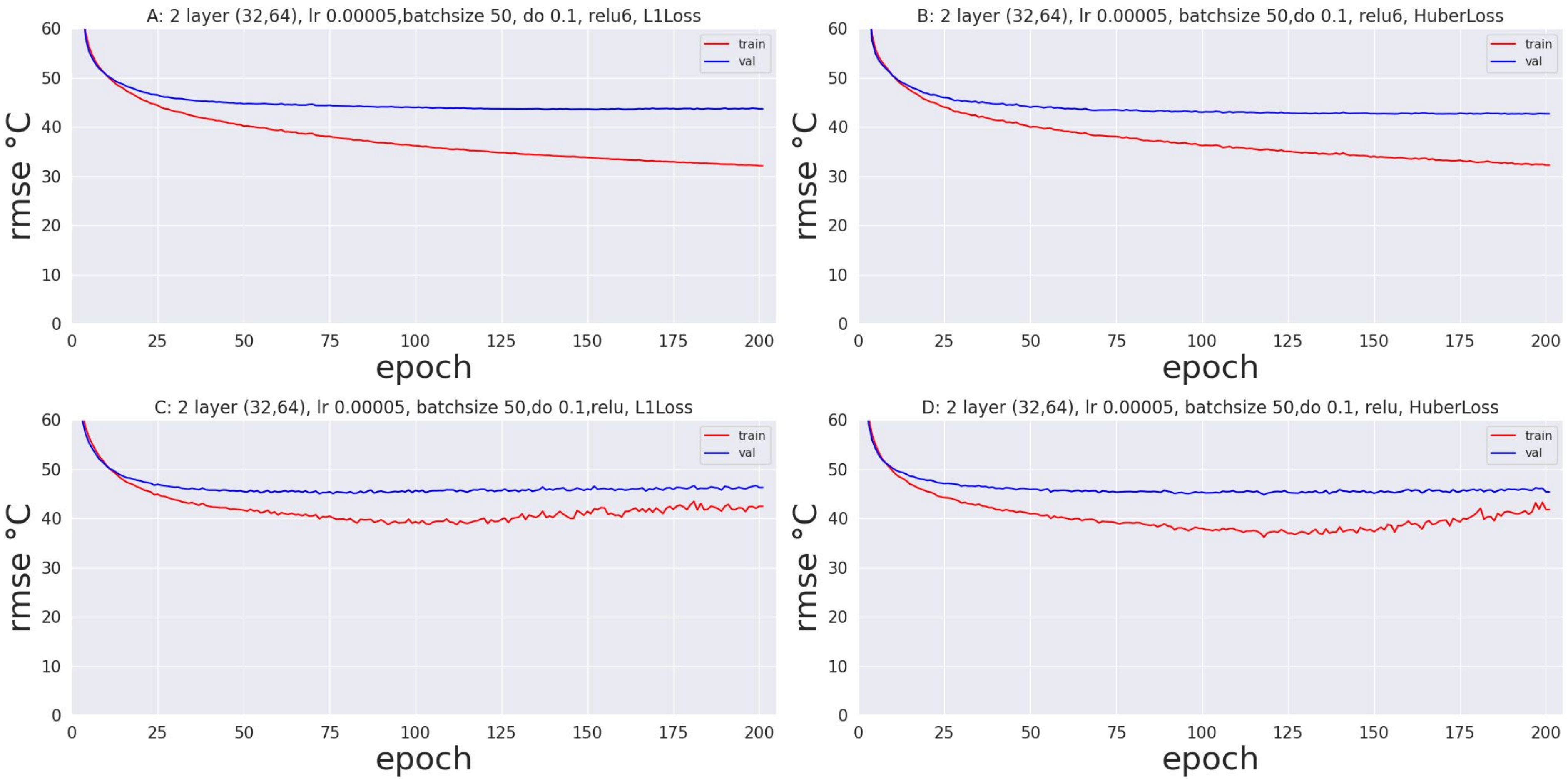

Figure S4. Plots of the *rmse* over the epochs to select the optimal parameters for the training of the neural networks for training set (red) and validation set (blue). Two hidden layers with 32 and 64 neurons are included, the dropout is set to 0.1 and a batch size of 50 is used, the lr is 0.00005, loss and activation function are varied, in (A) and (B) a ReLU6 activation function is applied, in (C) and (D) a ReLU function is applied. In (A) and (C) the L1Loss function is used, in (B) and (D) the HuberLoss function is used. In S1 and S2, the L2Loss function was applied.

SI3 Hyperparameter optimization of the GNN models for training with data augmentation applied.

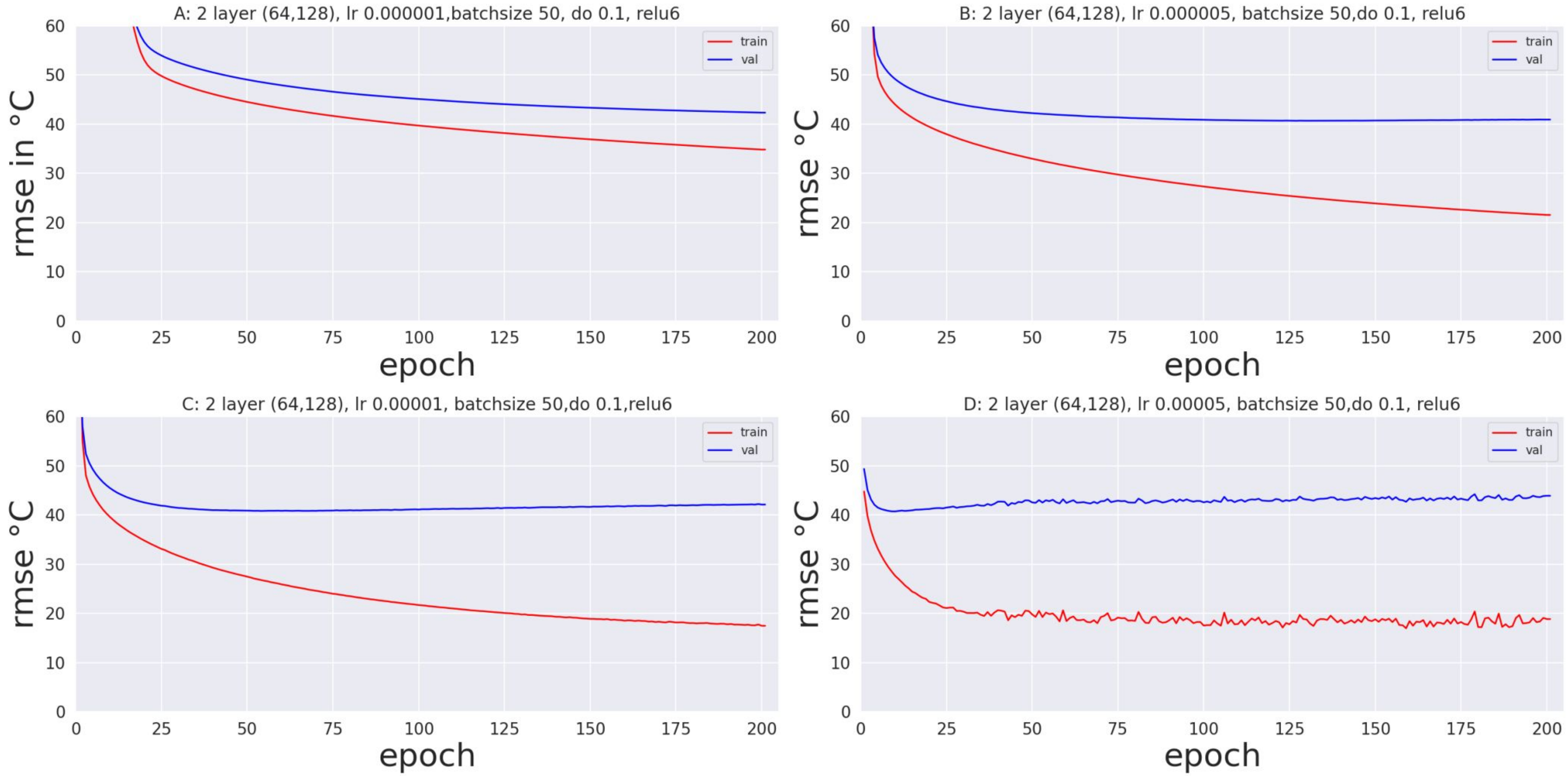

Figure S5. Plots of the *rmse* over the epochs to select the optimal parameters for the training of the neural networks for training set (red) and validation set (blue). Two hidden layers with 64 and 128 neurons are included, a ReLU6 activation function is applied, the dropout is set to 0.1 and a batch size of 50 is used. The learning rate (lr) is varied: (A) lr=0.000001, (B) lr=0.000005, (C) lr=0.00001, and (D) lr=0.00005.

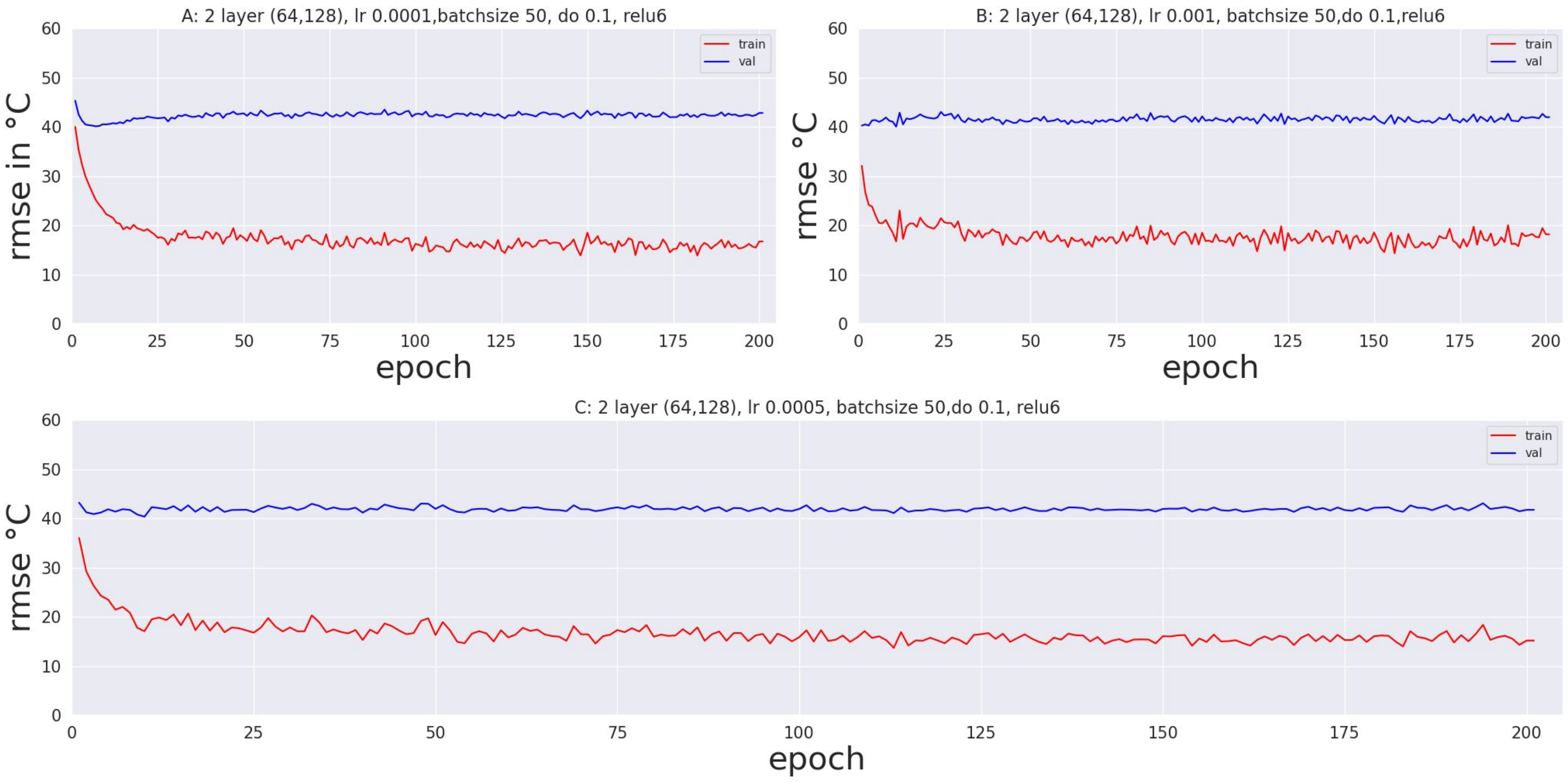

Figure S6. Plots of the *rmse* over the epochs to select the optimal parameters for the training of the neural networks for training set (red) and validation set (blue). Two hidden layers with 64 and 128 neurons are included, a ReLU6 activation function is applied, the dropout is set to 0.1 and a batch size of 50 is used. The learning rate (lr) is varied: (A) lr=0.0001, (B) lr=0.001, and (C) lr=0.0005.

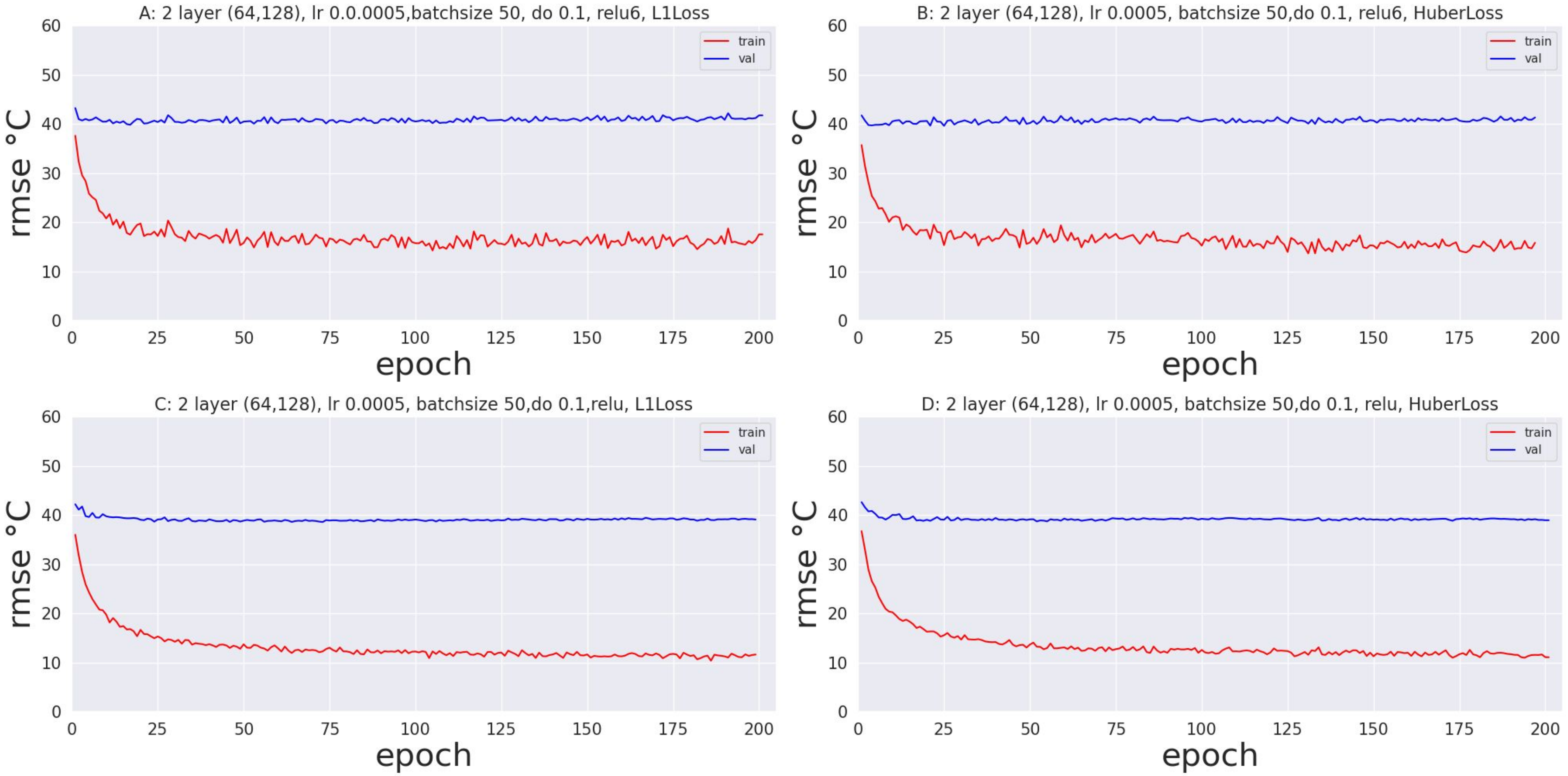

Figure S7. Plots of the *rmse* over the epochs to select the optimal parameters for the training of the neural networks for training set (red) and validation set (blue). Two hidden layers with 64 and 128 neurons are included, the dropout is set to 0.1 and a batch size of 50 is used, the lr is 0.00005, loss and activation function are varied, in (A) and (B) a ReLU6 activation function is applied, in (C) and (D) a ReLU function is applied. In (A) and (C) the L1Loss function is used, in (B) and (D) the HuberLoss function is used. In S1 and S2, the L2Loss function was applied.

SI4 Hyperparameter setup for the final nets including a comparison of the differences regarding the topology.

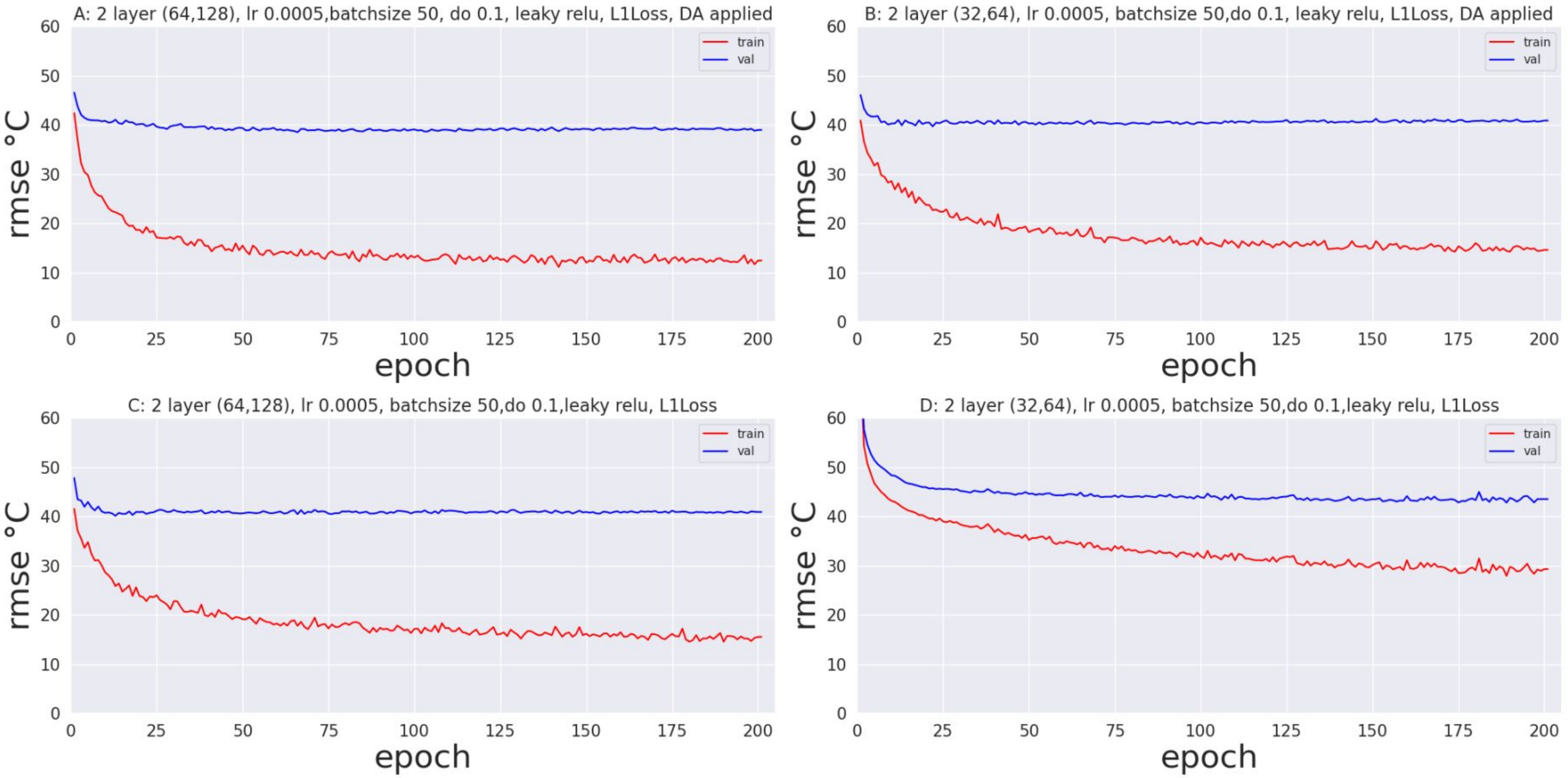

Figure S8. Plots of the *rmse* over the epochs to select the optimal parameters for the training of the neural networks for training set (red) and validation set (blue). Two hidden layers with 64 and 128 neurons are included for (A) and (C) and 32 and 64 neurons are included in (B) and (D). The dropout is set to 0.1 and a batch size of 50 is used, the lr is 0.0005, a L1Loss function and a leaky ReLU activation function is applied. In (A) and (B) the training set, where we applied data augmentation is used, in (C) and (D) the training set, where we did not apply data augmentation is used.

SI5 Evaluation of the impact of the topology applied in the different GNN models on the overall performance.

We tested whether the topology of the GNN models has an impact on the overall performance. As we selected 32 and 64 neurons in the two layers for models, where we did not apply data augmentation and 64 and 128 neurons for models, where we applied data augmentation, we wanted to check if the increased performance is related to the different structures of the GNNs. We selected a lower number of neurons for the models, where we did not apply data augmentation to avoid over parametrization. We again trained ten different GNN models and determined the corresponding statistical parameters for the consensus model. For the developed consensus model, the r^2 value was 0.827 and the corresponding q^2 value 0.793. The maximum negative error was -244.41°C and the maximum positive error was 215.76°C, we determined a bias of 1.59. The *rmse* was 37.45°C, which is slightly better compared to the results from the consensus model with 32 and 64 neurons. Still the performance is worse compared to the model, where we applied data augmentation. Thus, the topology does not impact the overall performance. We, therefore, decided to keep the different topologies, as we want to avoid overparameterization.

SI6 Prediction performance of the different consensus GNNs.

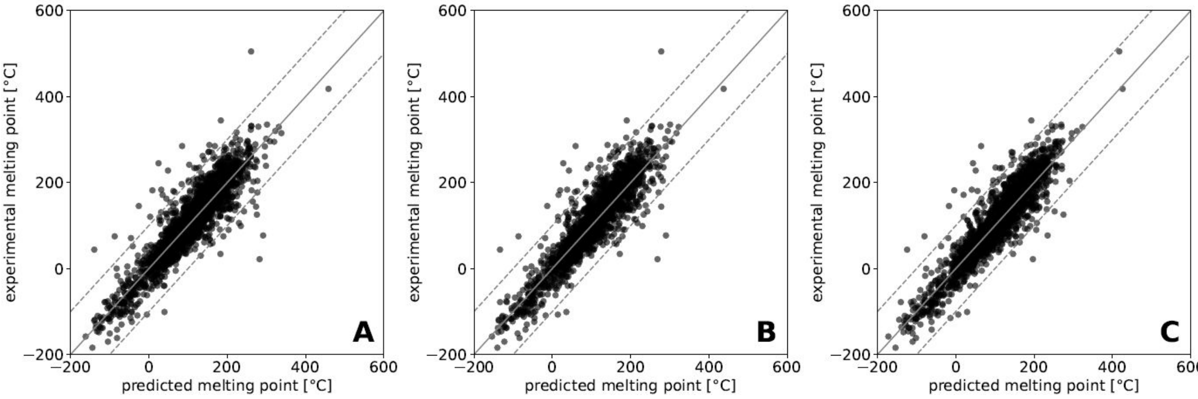

Figure S9. Plots of the experimental melting points versus predicted melting points for the consensus models trained on the initial (A) and curated (B) datasets without data augmentation and the initial dataset (C) where data augmentation was applied.
